# A Single Session of Temporomandibular Joint Soft Tissue Therapy and Its Effect on Pelvic Floor Muscles Activity in Women—A Randomized Controlled Trial

**DOI:** 10.3390/jcm13237037

**Published:** 2024-11-21

**Authors:** Iwona Sulowska-Daszyk, Sara Gamrot, Paulina Handzlik-Waszkiewicz

**Affiliations:** 1 Institute of Clinical Rehabilitation, University of Physical Education in Kraków, 31-571 Kraków, Poland; sara.gamrot@awf.krakow.pl; 2 Institute of Basic Sciences, University of Physical Education in Kraków, 31-571 Kraków, Poland; paulina.handzlik@awf.krakow.pl

**Keywords:** temporomandibular joint, pelvic floor muscles, EMG, muscle activity

## Abstract

**Background/Objectives**: Pelvic floor muscles (PFM) play a vital role in the proper functioning of the pelvic and abdominal organs. The PFM are structurally connected to other areas of the body, forming part of the deep front line. Due to its course, this line connects the PFM with the temporomandibular joint (TMJ). The aim of the study was to evaluate the impact of a single 15-minute soft tissue therapy session in the TMJ on the activity of the PFM. **Methods**: A total of 47 nulliparous women aged 20–29 years old diagnosed with myofascial pain in the TMJ area were included in the study. PFM were assessed using the Noraxon Ultium device and a vaginal probe, utilizing the surface electromyography (sEMG) method. The sEMG signal was processed with MyoResearch XP software version 1.0. Additionally, bladder floor displacement during PFM contractions was evaluated using an ultrasound imaging device set in B-mode (LOGIQ P7/P9). **Results**: In the experimental group, following the applied soft tissue therapy, a significant decrease in resting PFM activity between maximal contractions was observed (*p* < 0.05). The resting PFM activity assessed in the final phase of the measurement protocol was also significantly lower (*p* < 0.05). During endurance contractions in the experimental group, after the therapy, an 18.05% increase in PFM tension amplitude was noted, although this change was not statistically significant. In the control group, a decrease in amplitude was observed during the second assessment in this phase of the test. **Conclusions**: A single session of soft tissue therapy in the TMJ area may enhance the ability of the pelvic floor muscles to relax and contribute to improved muscle function by increasing their activation levels during submaximal contractions

## 1. Introduction

Pelvic floor muscles (PFM) play a vital role in the proper functioning of the pelvic and abdominal organs. The correct function of these muscles ensures continence of the urethra, vagina, and rectum while also supporting the internal organs of the abdomen and pelvis. Furthermore, the ability to appropriately contract and relax the PFM is crucial in sexual function and during childbirth [1,2].

The PFM are structurally connected to other areas of the body, forming part of the deep front line. Myofascial lines are defined as direct connections between adjacent muscle structures within the fascial network, transmitting tensions and overloads along these pathways. The deep front line (DFL) starts at the soles of the feet, passes along the back of the lower legs and knee, then moves through the inner thighs. It divides into two pathways: one runs through the front hip joint and lumbar spine, while the other goes along the posterior femur toward the pelvic floor, where they merge. From here, it continues through the thorax, surrounding internal organs and connecting to both the cranium and jaw. Through this pathway, the line connects the pelvic floor muscles to two distant regions—the soles of the feet and the temporomandibular joint (TMJ) [3].

The fascia surrounds muscles, organs, and bones and connects with various systems. It exhibits nociception and proprioception and is essential for functional integration. The concept of fascial communication suggests that anatomical fascial connections transmit pain and other effects resulting from dysfunctions in different parts of the musculoskeletal system. However, although the anatomy of the abdomen, lumbar spine, pelvis, and other structures is well described in the literature, there is a lack of data regarding the functional association of these structures that could explain the transmission of pain and effects associated with dysfunction [4,5].

Nowadays, a significant portion of the population experiences symptoms of TMJ dysfunction [6,7]. The prevalence of stomatognathic system dysfunction is often attributed to negative stress associated with modern living. Epidemiological data on the incidence of TMJ disorders vary widely [8]. Women are four times more likely to report TMJ dysfunctions, which, according to researchers, is due to hormonal factors and sex-related biological characteristics. The causes of these disorders are diverse and include structural factors (e.g., muscular, skeletal, neural), functional factors (e.g., lifestyle, posture), psychological factors (e.g., stress), or a combination of these [9]. Stress is a major factor in inducing muscle tension in various parts of the body. Research has shown that stress is associated with changes in the symmetry of the masticatory muscles, potentially leading to TMJ disorders [10]. Moreover, researchers have pointed to PFM hyperactivity as a chronic response to stress [11]. Prolonged excessive tension in the TMJ area leads to adaptive changes throughout the musculoskeletal system, resulting in altered posture, modifications in body biomechanics, and a disruption of overall body balance [12,13]. Many authors argue that, due to the biomechanical connections between the TMJ and other anatomical structures of the head, neck, shoulder girdle, and the broader musculoskeletal system, an interdisciplinary approach is essential for the treatment of TMJ dysfunction [13].

Several studies have confirmed clinical, anatomical, and functional connections between the cranial region and the pelvic floor muscles. Research suggests a positive correlation between the presence of bruxism and pain during sexual intercourse, which may be linked to increased muscle tension in both the jaw and pelvic regions [14]. Furthermore, a relationship has been described between the presence of pain on the right and left sides of the pelvis and pain on the right and left sides of the TMJ in women with endometriosis [15]. However, the mechanism of this relationship remains unclear, and the correlation between TMJ dysfunction and the neuromuscular activity of the PFM remains controversial. Understanding the complex interactions between the stomatognathic system and the PFM is crucial for achieving faster and more effective therapeutic outcomes in patients with PFM and TMJ dysfunctions [14,15,16].

A review of the literature reveals that, to date, the impact of TMJ therapy on PFM function has not been explored. So far, researchers have focused on the direct effects of soft tissue therapy either in the TMJ area [17,18,19] or exclusively on the PFM area [20,21]. To the authors’ knowledge, the indirect impact of soft tissue therapy in the TMJ area on PFM function has not yet been investigated. Given the functional and structural connections within the musculoskeletal system, it is reasonable to hypothesize that such therapy may yield beneficial effects on the PFM. The objective of this study was to evaluate the effect of a single session of temporomandibular joint soft tissue therapy on pelvic floor muscle activity.

## 2. Materials and Methods

### 2.1. Study Design

The study was conducted in the Functional Diagnostics Laboratory of the Central Scientific Research Laboratory at the University School of Physical Education in Kraków. It was a single-blind, randomized controlled trial conducted as part of a larger research project in accordance with the guidelines of the Declaration of Helsinki. Ethical approval for the study was obtained from the Bioethics Committee, approval number KBKA/50/O/2023. The project was funded under the Ministry of Science and Higher Education program ‘Regional Initiative of Excellence’ for the years 2019–2022, project number 022/RID/2018/19, with total funding of 11,919,908 PLN. The study was registered on the Australian New Zealand Clinical Trials Registry platform (registration ID: ACTRN12623000766617). The study followed the CONSORT guideline for randomized controlled trials.

### 2.2. Participants

The study included 47 women aged 20–29 years old diagnosed with myofascial pain in the TMJ area. The participants’ body weights ranged from 43 to 76.5 kg, and their heights ranged from 152 to 180 cm. Initial qualification of participants was based on the following inclusion criteria: age between 20 and 30 years old, nulliparity, absence of pregnancy and miscarriages, consent to participate in the study, a certificate confirming the absence of urogenital system disorders and approval for participation in the study issued by a gynecologist, and presence of symptoms in the TMJ area diagnosed by a maxillofacial surgeon in accordance with Diagnostic Criteria for Temporomandibular Disorders (DC/TMD): pain in the jaw, temple, in the ear, or in front of ear; pain modified with jaw movement, function or parafunction; confirmation of pain location in the temporalis or masseter muscle during the examination; report of familiar pain with palpation of the temporalis or masseter muscle; or report of pain spreading beyond the site of palpation but within the boundary of the muscle [22].

**Figure 1:**
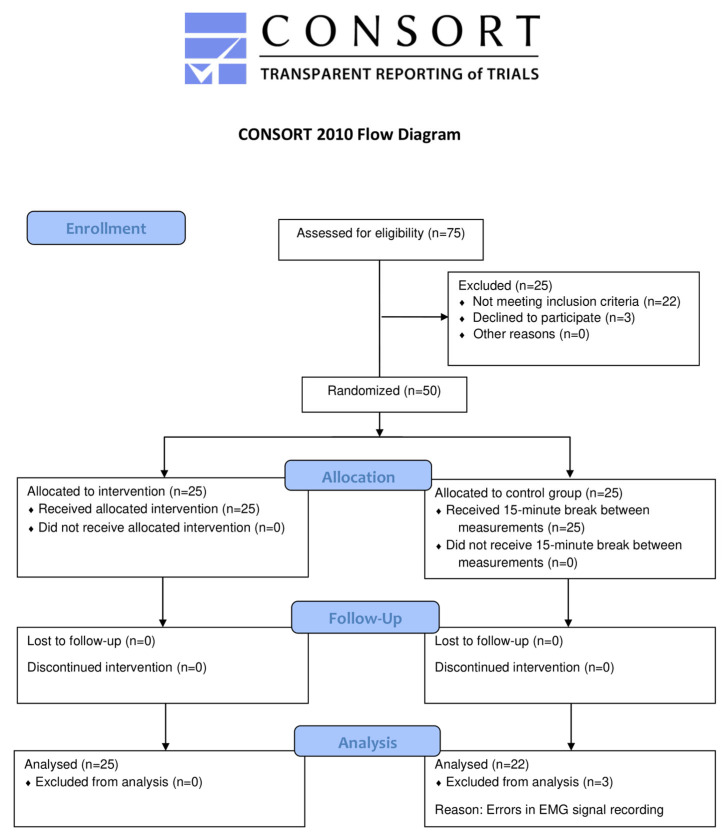
CONSORT flow diagram.

Exclusion criteria were as follows: pregnancy; neurological, systemic, or mental disorders; recent orthopedic procedures; urinary incontinence; prolapse of pelvic organs; vulvodynia; vestibulodynia; painful menstruation; endometriosis; surgeries involving reproductive organs; vaginal and bladder infections; injuries within the last 6 months prior to the study; and participation in TMJ therapy within the last 6 months. Recruitment of female participants took place from 22 September to 27 October 2023 through a social media announcement, which allowed us to reach a wide audience and conduct the initial recruitment process efficiently. All measurements were completed by 9 November 2023. The study flow diagram is presented in Figure 1.

This study was a parallel-group, randomized, controlled trial with a 1:1 allocation ratio. Participants were randomly divided into two groups using a computer-generated random number generator [23] by a member of the research team who did not participate in the patient recruitment process. The first group (n = 25) received a single 15-minute session of TMJ soft tissue therapy. The second group (n = 22) served as the control group with no intervention. The researcher who performed the measurements was blinded to the participant group allocation. Before commencing the study, participants were informed about the purpose and procedure of the research and therapy, and they provided written consent to participate in the research project. Each participant completed a custom questionnaire before the study, including questions about age, place of residence, education, as well as closed-ended questions regarding TMJ and pelvic floor dysfunctions. A detailed characterization of the study group is provided in Table 1.

To assess the effects of the applied therapy, measurements were taken twice: before and immediately after the 15-minute TMJ therapy session (in the first group), and measurements were taken twice with a 15-minute break between measurements without any intervention (in the second group). Before the commencement of the study, each patient was educated on the anatomy and function of the pelvic floor muscles and was provided with instructions on the correct activation of the PFM.

### 2.3. Research Tools

To evaluate the effectiveness of the applied therapy, measurements of PFM activity were conducted using research tools such as the Noraxon Ultium device (Noraxon U.S.A. Inc., Scottsdale, AZ, USA) and ultrasound imaging device set to B-mode (LOGIQ P7/P9, GE Healthcare, Seongnam, Republic of Korea).

#### 2.3.1. Ultrasonography

Ultrasonographic measurements of PFM function through the abdominal wall were performed using an ultrasound imaging device set to B-mode (LOGIQ P7/P9, GE Healthcare, Seongnam, Republic of Korea) with a 5 MHz convex transducer. Before imaging, a standardized bladder filling protocol was applied. The women were asked to fill their bladders by drinking 600–750 mL of water one hour before the measurements. The ultrasound measurement was performed in a supine position with a pillow under the head. The hips and knees were flexed and supported by a pillow under the knees, while the lumbar spine was in a neutral position. The ultrasound transducer was placed transversely across the midline of the abdomen, directly above the pubic symphysis. The probe angle was adjusted to approximately 60° from vertical and directed caudally or the posterior-inferior region of the bladder until a clear image of the bladder was obtained. The participants performed a series of PFM contractions before the recording to ensure the correct technique and proper placement and angling of the ultrasound transducer. Two screens were captured without removing the probe. During the first measurement, the patient was instructed to remain relaxed in order to image the position of the bladder during PFM relaxation. The subject was then asked to perform a maximal voluntary PFM contraction, and the image was captured at the point of maximum displacement. The ultrasound transducer was not moved during the procedure to ensure the field of vision remained constant between rest and maximal contraction. The level of PFM activation was assessed based on the displacement of the lower bladder wall following PFM contraction [24].

#### 2.3.2. Surface Electromyography (sEMG)

**Figure 2:**
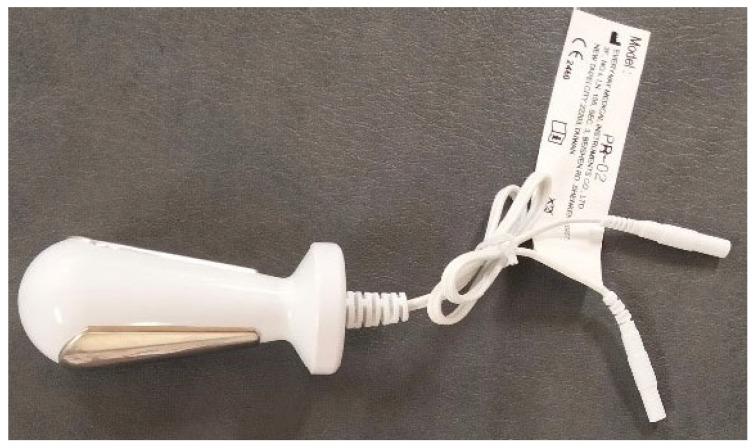
The Lifecare PR-02 vaginal probe (Everyway Medical Instruments Co., Ltd., Taiwan).

Surface electromyography (sEMG) was used to evaluate the PFM bioelectrical activity, using the Noraxon Ultium device (Noraxon U.S.A. Inc., Scottsdale, AZ, USA) and the Lifecare PR-02 vaginal probe (Everyway Medical Instruments Co., Ltd., New Taipei City, Taiwan). Studies indicate that various probe placements during functional PFM contraction did not have an impact on the outcomes of the sEMG assessments [25]. The signal was recorded with 16-bit resolution at a sampling frequency of 1500 Hz. sEMG signal processing was performed using the MyoResearch XP software version 1.0 (Noraxon USA, Inc., Scottsdale, AZ, USA). The vaginal probe, measuring 76 mm in length and 28 mm in diameter, featured two longitudinal stainless steel and nickel plates (Figure 2).

The study was not conducted during menstrual bleeding. Prior to the measurement, the patient was asked to empty her bladder. Then, after donning gloves and applying gel, the patient inserted the probe independently, positioning the plates toward the hips. The physiotherapist verified the correct placement of the electrode. The examination was performed in a supine position, with a bolster placed under the knees to ensure slight flexion of the hip and knee joints. The spine was maintained in a neutral position, and the upper limbs were aligned alongside the torso.

The study was conducted in accordance with the protocol proposed by Oleksy et al. [26], which included five activities:

A 60 s rest period (before the start of the examination—divided into three intervals: I—5 seconds, II—5 seconds, III—50 seconds)—participants were instructed to relax their PFM.

Five 2-second phasic contractions (quick contractions) with 10-second intervals—participants were instructed to contract their PFM as quickly as possible, then immediately and fully relax them.

Five 10-second tonic contractions with 10-second intervals—participants were instructed to contract their PFM as strongly as possible, hold the contraction for 10 seconds, and then fully relax.

One 60-second endurance contraction—participants were instructed to contract their PFM at a submaximal level that they could sustain for 60 seconds without altering the tension level.

A 60-second rest period (post-exercise)—participants were instructed to relax their PFM.

Additionally, the amplitude of the signal during the maximal voluntary contraction (MVC) of the PFM was measured to normalize the EMG signal.

### 2.4. Intervention

The therapy comprised soft tissue mobilization techniques both internally and externally of the temporomandibular joints (TMJ). A qualified therapist conducted the soft tissue therapy, with a duration of 15 minutes for bilateral treatment. The patient was positioned lying on their back, upper limbs aligned along the torso, head in a neutral position, and a therapeutic roll placed under the knees. The participants’ therapy was conducted by an experienced physiotherapist with many years of experience. External soft tissue release techniques included the following steps:

(A) Temporalis muscle relaxation:
  - Trigger point therapy on the temporalis muscle (technique duration: 1 minute).
  - Myofascial release along the path of the trigger point on the temporalis muscle (technique duration: 1 minute).
  - Positional relaxation of the temporalis muscle (technique duration: 30 seconds).
  - Myofascial release of the temporalis muscle; work along the muscle from top to bottom (technique duration: 30 s).
(B) Masseter muscle relaxation: myofascial release along the path of the muscle from top to bottom (technique duration: 30 s).

Internal work included the following steps:

(A) Pterygoid muscle relaxation (technique duration: 1 minute).
(B) Masseter muscle relaxation:
  - Myofascial release along the path of the muscle fibers (technique duration: 1 minute).
  - Trigger point release in the attachment area (technique duration: 1 minute).

**Figure 3:**
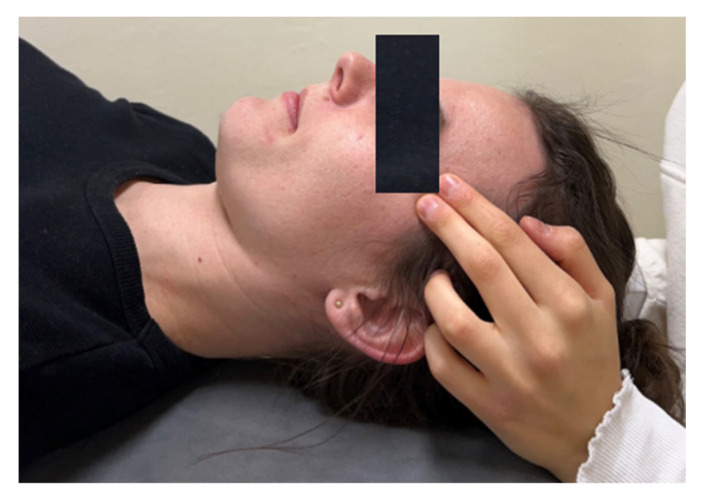
Temporalis muscle relaxation—trigger point therapy, external technique.

**Figure 4:**
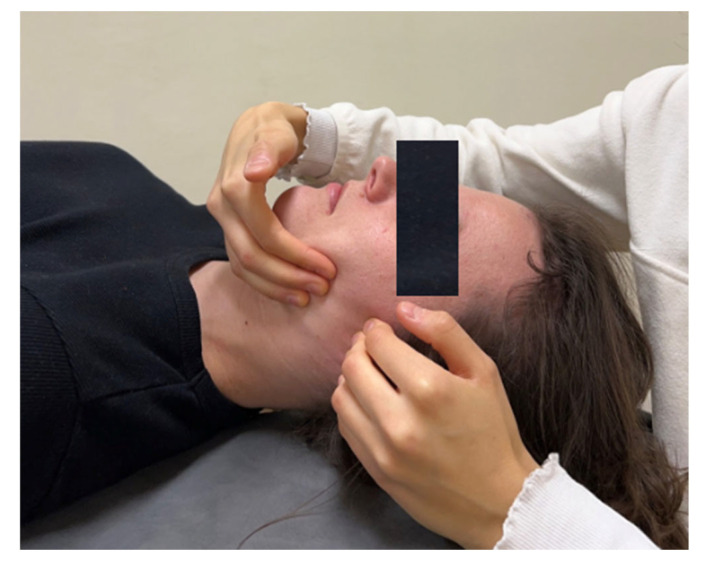
Masseter muscle relaxation: myofascial release, external technique.

**Figure 5:**
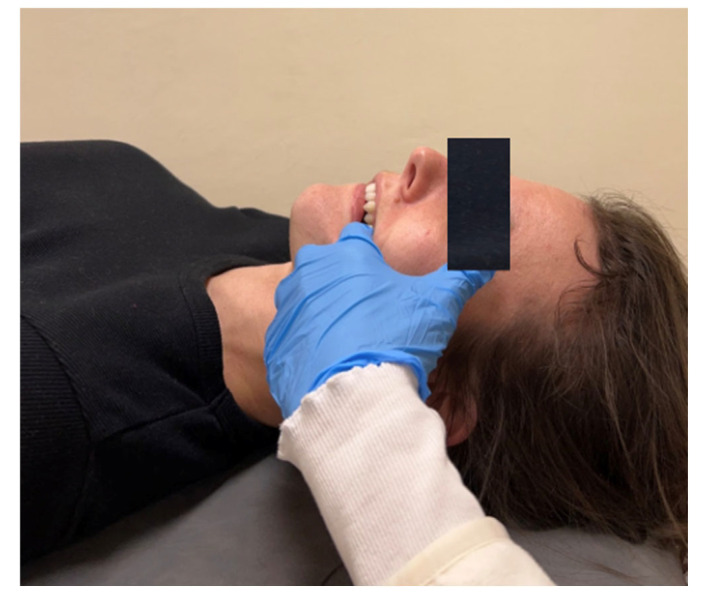
Pterygoid muscle relaxation—internal technique.

**Figure 6:**
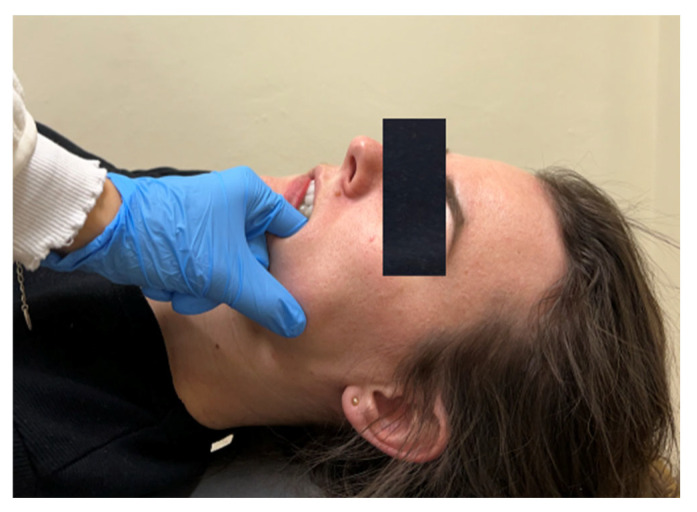
Masseter muscle relaxation—trigger point release in the attachment area, internal technique.

The example techniques are presented in Figure 3, Figure 4, Figure 5 and Figure 6.

### 2.5. Statistical Analysis

The statistical analysis was conducted using STATISTICA 12.0 Pl (Statsoft Polska, Krakow, Polska). The normality of variable distribution was assessed using the Shapiro–Wilk test. To evaluate the significance of differences in the variables studied, a two-way analysis of variance (ANOVA) was performed with one main factor between groups (experimental and control) and the other main factor being the repeated measure (time: before and after therapy in the experimental group and before and after a 15-minute break in the control group). Tukey’s post-hoc test was used for pairwise comparisons. The effect size was calculated using Cohen’s d and interpreted as negligible (<0.1), small (0.1–0.3), medium (0.3–0.5), and large (>0.5) [27]. Differences were considered statistically significant if the test probability level was lower than the assumed significance level (*p* < 0.05). Power analysis for two-way ANOVA allowed the study to determine that at least 16 participants from each group were required to obtain a power of 0.8 at a two-sided level of 0.05 with the effect size of d = 0.8. This analysis was based on data derived from previous literature on the primary outcome variables such as PFM bioelectrical activity [28]. An analysis based on another study evaluating the effectiveness of TMJ therapy on jaw mobility determined that at least 16 participants from each group were required to obtain assumed power [29].

## 3. Results

### 3.1. Activation of the PFM Using USG Measurement

Following a single session of TMJ therapy in the experimental group, an increase in pelvic floor muscle activation by 52.94% was observed. The calculated effect size indicated a large effect. In the control group, the level of PFM activation increased by 8.89%. However, this change was not statistically significant in either group. The data are presented in Table 2.

### 3.2. Bioelectrical Activity of the PFM

#### 3.2.1. Resting Activity of the PFM During a 1-Minute Rest Period (First Phase of the Measurement Protocol—Pre-Exercise)

In the experimental group, no significant changes in pre-exercise resting activity were observed after the applied therapy. In the control group, resting activity significantly decreased in the second assessment (*p* < 0.05). Significant differences were also observed in the second assessment in the between-group comparison. The results are presented in Table 3.

#### 3.2.2. Activity of the PFM During 2-Second Phasic Contractions

In the next phase of the applied protocol, PFM activity during 2-second phasic contractions was assessed. No significant changes in PFM activity amplitude during contraction were observed in either group. However, in the experimental group, after the applied therapy, a reduction in resting PFM activity between phasic contractions was noted. This change was statistically significant (*p* < 0.05). The data are presented in Table 4.

#### 3.2.3. Activity of the PFM During 10-Second Tonic Contractions

During the assessment of 10-second tonic contractions, no significant changes were observed in either group (Table 5).

#### 3.2.4. Activity of the PFM During 60-Second Endurance Contraction

In the experimental group, during endurance contractions following the applied therapy, an increase in PFM activity amplitude of 18.05% was observed, although this change was not statistically significant (*p* = 0.197). In the control group, a decrease in signal amplitude of 12.03% was noted. In both groups, a reduction in the muscle fatigue index (f ratio) was observed in the second assessment—4.68% in the experimental group and 1.41% in the control group. The data are presented in Table 6.

#### 3.2.5. Resting Activity of the PFM During a 1-Minute Rest Period (Last Phase of the Measurement Protocol—Post-Exercise)

During the 1-minute rest phase in the last stage of the measurement protocol, a significant reduction in resting PFM activity was observed after the applied TMJ therapy (*p* < 0.05). No significant changes were noted in the control group in the second assessment (Table 7).

## 4. Discussion

This study examined the impact of a single session of temporomandibular joint (TMJ) soft tissue therapy on pelvic floor muscles (PFM) activity. The most novel finding of this research is that the applied therapy significantly enhanced the ability of the pelvic floor muscles to relax. An increase in PFM activation levels during endurance contractions was also observed. To the authors’ knowledge, this study is the first to explore the effects of a single TMJ therapy session on PFM activity.

Other studies confirm the connection between the cranial region and the pelvic floor muscles. These muscles are part of the deep front line, which, due to its path, links the PFM with the TMJ [3]. Fischer et al. [30] suggested that TMJ dysfunction may significantly limit hip joint mobility in patients with complex regional pain syndrome (CRPS), indicating a biomechanical connection between these two regions. The study conducted by Minguez-Esteban et al. [14] indicates a correlation between bruxism and painful intercourse. The authors hypothesize that the observed relationship results from increased muscle tension in both areas. However, they emphasize that the underlying mechanism behind this association remains unclear and requires further investigation. Research conducted by Wójcik et al. [15] among women diagnosed with endometriosis has shown a correlation between pain on the right and left sides of the pelvis and pain in the right and left sides of the TMJ. However, the authors point out that pain-related factors may be relevant in interpreting the study’s findings. Patients may experience mixed pain, which is defined as a complex overlap of different known types of pain (nociceptive and neuropathic) in any combination, acting simultaneously and/or concurrently. The modern theory of pain views it as a multifaceted process in which pain is not just a result of tissue damage but also a brain response to these signals [31].

Many authors describe the use of sEMG as an easy and non-invasive method for assessing PFM [26,32]. Analyzing the EMG results from our study, a significant decrease in resting activity between maximal PFM contractions was observed in the experimental group during the second measurement. This suggests that the applied TMJ soft tissue therapy may have improved the ability of the PFM to relax between fast maximal contractions. However, the present study did not assess the effects of the therapy on other muscle groups’ activity. Therefore, the observed outcomes might have been attributed to a generalized relaxation effect across the entire body rather than localized impacts on the pelvic floor muscles alone.

After therapy, resting PFM activity assessed in the final phase of the measurement protocol was also significantly lower. This may indicate a relaxing effect of TMJ therapy on the PFM, which, despite fatigue from the phasic, tonic, and endurance contractions, showed greater relaxation in the second measurement. However, this trend was not observed in the assessment of resting activity conducted at the beginning of the protocol. At this stage, a significant decrease in resting PFM activity was noted in the control group when comparing the first and second measurements. This result is intriguing. It is possible that this effect was caused by the 15-minute rest in a lying position between measurements, but further research is certainly needed to clarify these results.

It is hypothesized that performing PFM contractions is a relaxation technique that reduces vaginal pressure and resting PFM activity [33]. Our study seems to support this claim. Comparing the resting PFM activity from the initial and final phases of the protocol in the first measurement, a decrease in muscle activity was observed by 8.39% in the experimental group and 15.12% in the control group after completing all protocol phases. However, the results from the second measurement, where the experimental group underwent TMJ soft tissue therapy and the control group had a 15-minute rest, showed different outcomes. Comparing the initial and final resting phases, a significant decrease in resting PFM activity of 24.90% was observed in the experimental group. This result may also suggest that the applied therapy increased the PFM’s relaxation capacity. In contrast, the control group showed an increase in resting PFM activity of 16.51% during the final phase of the measurement protocol.

There are few studies that evaluate the impact of a single PFM therapy session on the activity of these muscles, and their results are inconclusive. Research conducted by Kasper-Jędrzejewska et al. [20] among women with urinary incontinence indicates that a one-time soft tissue therapy intervention on the PFM did not lead to a significant change in the electromyographic signal of these muscles. On the other hand, according to Błudnicka et al. [34], even one session of EMG biofeedback applied to pregnant women significantly increases PFM contraction awareness, improving the firing order in the PFM contractions. Additionally, according to the same authors, a single session of EMG biofeedback therapy increases the amplitude of the PFM signal [28].

So far, a reduction in resting activity of the PFM has been observed following various multi-week PFM training programs [35,36]. Multi-week manual therapy targeting the pelvic floor, aimed at reducing excessive muscle tension, has been shown to effectively alleviate symptoms of overactive bladder syndrome and interstitial cystitis. In electromyographic assessment, the average resting activity of the PFM decreased by 65% [21].

Manual therapy enhances muscle and fascial elasticity, reduces pain, regulates the nervous system by lowering excessive sympathetic activity, and improves coordination between agonist and antagonist muscles [37]. In our study in the experimental group, during endurance contractions following the applied therapy, an increase in PFM activity amplitude by 18.05% was observed. However, this change was not statistically significant. In the control group, a decrease in amplitude was noted in the second measurement. During tonic contractions in the second measurement, both groups experienced a slight increase in PFM amplitude. It can be assumed that the relaxation of tissues around the TMJ reduced the resting activity of the PFM, improving the ability for more precise and controlled contractions. This effect was not observed in the evaluation of maximal contractions, where both groups showed slightly lower amplitude values in the second test. These results suggest that a single session of TMJ soft tissue therapy does not increase the maximum activation capacity of the muscles but rather enhances coordination, leading to improved performance in submaximal contractions. Similar results were obtained by Szumilewicz et al. [38], where a 6-week training program combining high and low-intensity aerobics with PFM exercises did not lead to an increase in their amplitude during a 1-minute submaximal contraction. However, a favorable trend in increased neuromuscular activity was observed during both the 1-minute contraction and 10-second tonic contractions, although these changes were not statistically significant.

In our study, one of the stages of the measurement protocol during the EMG assessment was the evaluation of the change in median frequency during the isometric submaximal contraction of the PFM. According to the literature, a shift in median frequency toward lower values indicates muscle fatigue [39]. The variable reflecting the motor endurance of the PFM was calculated as the ratio of the median frequency in the last 5 seconds of the 1-minute submaximal contraction to that in the middle 5 seconds. Due to the delayed activation of the PFM by some subjects, the calculation of the ratio of the last 5 seconds compared to the first 5 seconds was omitted, which would certainly have provided a more comprehensive picture of the change in median frequency throughout the entire minute-long contraction. Researchers indicate that in the analysis of this indicator, a greater decrease in median frequency signifies greater muscle fatigue [40].

Comparing the fatigue index of the PFM in the experimental group, slightly smaller decreases were observed in the second measurement (the index was 17.75 before therapy and 16.92 after therapy). However, this change was not statistically significant. A single session of therapy is certainly too weak a stimulus to enhance muscle endurance. Therefore, it would be worthwhile to assess in future studies the impact of prolonged TMJ therapy on the endurance of the PFM.

Another tool used to assess the function of the PFM is abdominal ultrasonography. This method relies on evaluating the movement of the base of the bladder as an indicator of PFM activity [41,42]. In this study, the experimental group exhibited a 52.94% increase in PFM activation during contraction after TMJ soft tissue therapy, while in the control group, this value increased by 8.89%. The results obtained in the experimental group were on the verge of statistical significance; however, the observed large effect size seems to indicate the positive impact of TMJ soft tissue therapy on PFM functions.

In another study [43], the impact of PFM exercises on strength was evaluated in women with urinary incontinence using abdominal ultrasonography. The intervention involved a 12-week training program in which the experimental group performed PFM exercises, and the effects were compared to a control group. The use of abdominal ultrasonography allowed for precise measurement of bladder base displacement during muscle contractions. The enhancement in all PFM strength parameters was significantly higher in the experimental group than in the control group after 12 weeks of treatment. The increase in PFM strength was 160.6% according to abdominal ultrasonography in the transverse plane and 169.4% according to abdominal ultrasonography in the longitudinal plane [43].

Hagovska et al. [44] also demonstrated that a 12-week training program led to significant improvement in symptoms of stress urinary incontinence (SUI) and the function of PFM, including better support and elevation of the bladder during PFM contractions using 3D/4D ultrasonography.

The pelvic floor muscles are considered part of the deep front line, which may explain the observed changes in PFM activity following TMJ soft tissue therapy [3]. Although the exact mechanism behind this connection remains unclear, the presented results contribute to the growing body of evidence suggesting that tension or dysfunction in one part of the fascial network can impact other, seemingly unrelated areas.

However, several limitations of this study should be noted. The observed effects could also be influenced by individual differences in muscle tension, stress levels, and baseline TMJ function, which were not fully controlled in this study. An important limitation of this study is the lack of data on the effects of the therapy on other muscle groups in the body, as only the pelvic floor muscles were measured. To clearly demonstrate a specific relationship between TMJ therapy and pelvic floor muscle function, it would be valuable to conduct an experiment confirming that the effects of TMJ treatment are not systemic and do not impact other muscle groups. It would also be valuable to assess the effects of TMJ therapy in women reporting dysfunctions in the genitourinary system.

From a clinical perspective, our study’s findings hold significant relevance. First, TMJ soft tissue therapy may enhance the pelvic floor muscles’ ability to relax and improve their function. Second, these results emphasize the importance of an integrated treatment approach, considering the connections between the TMJ and the pelvic floor. Future research should aim to replicate these measurements in larger study groups and explore potential mechanisms linking TMJ function with PFM activity. Additionally, they should assess the impact of therapy on women with genitourinary system dysfunctions. This could lead to new therapeutic approaches that integrate TMJ and PFM treatment in patients with dysfunctions in both areas, which affects the comfort and health of women.

## 5. Conclusions

The results obtained in this study suggest that a single session of temporomandibular joint soft tissue therapy significantly enhances the ability of the pelvic floor muscles to relax. It may also contribute to improving muscle function by increasing the level of activation during submaximal contractions. While this study focused on women without genitourinary dysfunctions, the findings raise the possibility of applying these TMJ therapeutic techniques in the treatment of PFM dysfunctions. Further research in this area is certainly needed to confirm these effects.

## Figures and Tables

**Table 1:** Characteristics of the participants.

	Experimental Group (n = 25) Mean ± SD	Control Group (n = 22) Mean ± SD
Age [years]	23.84 ± 1.97	23.50 ± 2.22
High [cm]	166.16 ± 5.71	166.45 ± 6.49
Body mass [kg]	58.06 ± 6.78	59.85 ± 9.06
BMI [kg/m^2^]	21.05 ± 2.46	21.59 ± 2.98

SD—standard deviation; cm—centimeters; kg—kilograms; BMI—body mass index; m—meters.

**Table 2:** Activation of the PFM using USG measurement.

	Measurement	Experimental Group (n = 25) Mean ± SD	*p*	ES ^a^	Control Group (n = 22) Mean ± SD	*p*	ES ^a^	*p* *	ES ^b^
Activation of the pelvic floor muscles [cm]	Before	0.34 ± 0.23	0.059	0.621	0.45 ± 0.34	0.990	0.099	0.203	0.379
	After	0.52 ± 0.34			0.49 ± 0.46			0.785	−0.074

SD—standard deviation; cm—centimeters; *p*—between measurements; *p* *—between groups; ES ^a^—effect size (Cohen’s d) within each group; ES ^b^—effect size (Cohen’s d) between study groups.

**Table 3:** Resting activity of the PFM during a 1-minute rest period (first phase of the measurement protocol—pre-exercise).

	Measurement	Experimental Group (n = 25)		*p*	ES ^a^	Control Group (n = 22)		*p*	ES ^a^	*p* *	ES ^b^
		Mean ± SD	%MVC			Mean ± SD	%MVC				
Average mean amplitude (µV)	Before	7.27 ± 3.99	16.74 ± 9.33	0.912	0.017	6.88 ± 3.94	15.49 ± 11.16	0.002	−0.756	0.752	−0.098
	After	7.35 ± 5.29	14.78 ± 7.59			4.30 ± 2.79	10.26 ± 7.94			0.014	−0.721

SD—standard deviation; *p*—between measurements; *p* *—between groups; ES ^a^—effect size (Cohen’s d) within each group; ES ^b^—effect size (Cohen’s d) between study groups; %MVC—percent of maximal voluntary contraction; µV—microvolts; %—percent.

**Table 4:** Activity of the PFM during 2-second phasic contractions.

	Measurement	Experimental Group (n = 25)		*p*	ES ^a^	Control Group (n = 22)		*p*	ES ^a^	*p* *	ES ^b^
		Mean ± SD	%MVC			Mean ± SD	%MVC				
Average peak amplitude (µV)	Before	46.11 ± 28.83	84.45± 7.82	0.555	−0.072	50.27 ± 36.88	83.87 ± 9.63	0.270	−0.118	0.661	0.126
	After	44.01 ± 29.48	72.34 ± 21.54			46.07 ± 34.04	78.10 ± 15.87			0.828	0.065
Time before peak (s)	Before	31.98 ± 12.02	-	0.130	0.315	32.93 ± 12.49	-	0.759	−0.071	0.813	0.078
	After	36.43 ± 15.99	-			31.99 ± 13.83	-			0.271	−0.297
Average resting tension between phasic contractions (µV)	Before	8.39 ± 5.20	17.31 ± 8.88	0.005	−0.430	6.43 ± 4.34	20.09 ± 17.60	0.136	−0.333	0.125	0.409
	After	6.27 ± 4.64	13.13 ± 9.07			5.26 ± 2.43	10.66 ± 6.91			0.426	−0.273

SD—standard deviation; *p*—between measurements; *p* *—between groups; ES ^a^—effect size (Cohen’s d) within each group; ES ^b^—effect size (Cohen’s d) between study groups; %MVC—percent of maximal voluntary contraction; µV—microvolts; %—percent; s—second.

**Table 5:** Activity of the PFM during 10-second tonic contractions.

	Measurement	Experimental Group (n = 25)		*p*	ES ^a^	Control Group (n = 22)		*p*	ES ^a^	*p* *	ES ^b^
		Mean ± SD	%MVC			Mean ± SD	%MVC				
Average mean amplitude (µV)	Before	21.86 ± 15.50	41.01 ± 11.81	0.756	0.036	23.28 ± 18.77	38.01 ± 14.61	0.834	0.020	0.784	0.082
	After	22.42 ± 15.81	38.19 ± 16.35			23.68 ± 20.42	37.31 ± 16.69			0.807	0.069
Time before peak (s)	Before	35.02 ± 19.14	-	0.375	−0.222	37.67 ± 18.41	-	0.522	−0.189	0.619	0.141
	After	30.80 ± 18.86	-			34.43 ± 15.81	-			0.497	0.209
Average resting tension between tonic contractions (µV)	Before	9.64 ± 6.11	19.28 ± 9.66	0.171	−0.213	9.37 ± 6.26	22.34 ± 14.53	0.051	−0.318	0.869	−0.044
	After	8.49 ± 4.59	17.10 ± 8.01			7.61 ± 4.70	15.45 ± 8.61			0.583	−0.189

SD—standard deviation; *p*—between measurements; *p* *—between groups; ES ^a^—effect size (Cohen’s d) within each group; ES ^b^—effect size (Cohen’s d) between study groups; %MVC—percent of maximal voluntary contraction; µV—microvolts; %—percent; s—second.

**Table 6:** Activity of the PFM during 60-second endurance contraction.

	Measurement	Experimental Group (n = 25)		*p*	ES ^a^	Control Group (n = 22)		*p*	ES ^a^	*p* *	ES ^b^
		Mean ± SD	%MVC			Mean ± SD	%MVC				
Average mean amplitude (µV)	Before	16.68 ± 11.32	33.52 ± 17.29	0.197	0.211	17.13 ± 14.62	31.50 ± 16.44	0.405	−0.165	0.910	0.034
	After	19.69 ± 16.70	31.05 ± 14.27			15.07 ± 9.89	27.99 ± 12.83			0.245	−0.337
Median frequency (Hz)	Before	61.24 ± 18.08	-	0.959	0.011	67.27 ± 25.09	-	0.507	0.129	0.393	0.276
	After	61.48 ± 25.81	-			70.60 ± 26.53	-			0.198	0.348
Mean frequency (Hz)	Before	80.95 ± 23.57	-	0.959	−0.009	89.88 ± 33.56	-	0.235	0.167	0.330	0.308
	After	80.71 ± 27.53	-			95.95 ± 38.86	-			0.098	0.453
F ratio	Before	17.75 ± 2.96	-	0.424	−0.257	16.28 ± 1.90	-	0.898	−0.101	0.083	0.591
	After	16.92 ± 3.48	-			16.05 ± 2.61	-			0.194	0.283

SD—standard deviation; *p*—between measurements; *p* *—between groups; ES ^a^—effect size (Cohen’s d) within each group; ES ^b^—effect size (Cohen’s d) between study groups; %MVC—percent of maximal voluntary contraction; µV—microvolts; %—percent; Hz—hertz, f ratio—ratio of the median frequency of the third interval to the second interval.

**Table 7:** Resting activity of the PFM during a 1-minute rest period (last phase of the measurement protocol—post-exercise).

	Measurement	Experimental Group (n = 25)		*p*	ES ^a^	Control Group (n = 22)		*p*	ES ^a^	*p* *	ES ^b^
		Mean ± SD	%MVC			Mean ± SD	%MVC				
Average mean amplitude (µV)	Before	6.66 ± 4.16	15.01± 9.55	0.028	−0.276	5.84 ± 3.72	16.25 ± 14.68	0.128	−0.247	0.461	−0.208
	After	5.52 ± 4.11	12.31 ± 7.74			5.01 ± 2.96	11.56 ± 9.25			0.645	−0.142

SD—standard deviation; *p*—between measurements; *p* *—between groups; ES ^a^—effect size (Cohen’s d) within each group; ES ^b^—effect size (Cohen’s d) between study groups; %MVC—percent of maximal voluntary contraction; µV—microvolts; %—percent.

## Data Availability

All data generated or analyzed during this study are included in this published article.
